# Training benchmarks based on validated composite scores for the RobotiX robot-assisted surgery simulator on basic tasks

**DOI:** 10.1007/s11701-020-01080-9

**Published:** 2020-04-20

**Authors:** Erik Leijte, Linda Claassen, Elke Arts, Ivo de Blaauw, Camiel Rosman, Sanne M. B. I. Botden

**Affiliations:** 1 Department of Surgery, Radboud University Medical Center, Geert Grooteplein 10 route 618, 6500HB Nijmegen, The Netherlands; 2 Department of Pediatric Surgery, Radboud University Medical Center, Nijmegen, The Netherlands

**Keywords:** Robot-assisted, Simulation, Validity evidence, Surgical education

## Abstract

The RobotiX robot-assisted virtual reality simulator aims to aid in the training of novice surgeons outside of the operating room. This study aimed to determine the validity evidence on multiple levels of the RobotiX simulator for basic skills. Participants were divided in either the novice, laparoscopic or robotic experienced group based on their minimally invasive surgical experience. Two basic tasks were performed: wristed manipulation (Task 1) and vessel energy dissection (Task 2). The performance scores and a questionnaire regarding the realism, didactic value, and usability were gathered (content). Composite scores (0–100), pass/fail values, and alternative benchmark scores were calculated. Twenty-seven novices, 21 laparoscopic, and 13 robotic experienced participants were recruited. Content validity evidence was scored positively overall. Statistically significant differences between novices and robotic experienced participants (construct) was found for movements left (Task 1 *p* = 0.009), movements right (Task 1 *p* = 0.009, Task 2 *p* = 0.021), path length left (Task 1 *p* = 0.020), and time (Task 1 *p* = 0.040, Task 2 *p* < 0.001). Composite scores were statistically significantly different between robotic experienced and novice participants for Task 1 (85.5 versus 77.1, *p* = 0.044) and Task 2 (80.6 versus 64.9, *p* = 0.001). The pass/fail score with false-positive/false-negative percentage resulted in a value of 75/100, 46/9.1% (Task 1) and 71/100, 39/7.0% (Task 2). Calculated benchmark scores resulted in a minority of novices passing multiple parameters. Validity evidence on multiple levels was assessed for two basic robot-assisted surgical simulation tasks. The calculated benchmark scores can be used for future surgical simulation training.

**Electronic supplementary material:**

The online version of this article (10.1007/s11701-020-01080-9) contains supplementary material, which is available to authorized users.

## Introduction

In recent years, the number of robotically assisted surgeries rose worldwide from 499,000 procedures in 2015 to 644,000 procedures in 2017. With these increasing numbers, a broader application of robot-assisted surgery was seen, showing growth mainly in general surgery procedures, such as hernia repair and colorectal procedures, according to the annual report 2017 of Intuitive Surgical Inc. [1]. These developments led to an increased demand for robotic training systems and curricula, to train both novice and experienced surgeons. The intra-operative learning method is not preferred in daily practice, as the robot-assisted intra-operative learning curve uses costly operating room time and material. Furthermore, intra-operative learning poses the ethical concern of practicing on patients at the cost of patient safety [2–5]. These undesirable aspects of intra-operative learning have stimulated a shift towards virtual reality simulation of robot-assisted surgery, which is already shown to be effective for minimal invasive surgery [3, 6–8]. Nowadays, different systems are available, each with a different setup and exercises, to simulate robotic surgery in a safe virtual reality setting. Currently, the most used systems are the Da Vinci Skills Simulator (Intuitive Surgical, Inc., Sunnyvale, CA), the Mimic dV-Trainer (Mimic Technologies, Inc., Seattle, WA), the Robot Surgical Simulator (Simulated Surgical Systems, LLC, Williamsville, NY), and the RobotiX Mentor (3D Systems Inc., Cleveland, OH). With the RobotiX mentor being the newest addition of the robot simulators, there are only a few studies performed investigating the validity of the system [9–15]. For the exercises of a simulator to be effectively used in a training curriculum, validation studies have to be performed to assess the value of each exercise in terms of realism, usability, and the capability to differentiate between expertise levels [16–18]. This study aims to assess the validity of the RobotiX for two separate basic tasks, using the relevant sources of validity according to Messick’s framework of validity (content, response process, relation to other variables, and consequences of the test) [19]. Valid outcome parameters will be processed to a composite score, which can be used for benchmarks during training of surgical residents and surgeons, still new to robot-assisted surgery. Besides the conventional expert versus novice comparison, a laparoscopic experience group was included as these are likely to be assessed on their robot-assisted skills as well, but already acquired some minimally invasive skills [20].

## Methods

### Participants

The participants were recruited at the Radboud University Medical Center Nijmegen, the Netherlands and during the European Association of Urology congress 2018. The subjects were divided into three groups based on their self-reported surgical experience. Subjects in the novice group had no clinical experience. All novice participants consisted of medical interns who understood the concept of laparoscopy and robot-assisted surgery. Subjects in the laparoscopic experience group had performed > 10 clinical laparoscopic procedures, without clinical robot-assisted surgery experience. This group was included to as they are most likely the first to start learning robot-assisted surgery. Subjects in the robotic experience group had performed > 10 basic robotic procedures in the clinical setting and were not previously trained on the RobotiX simulator.

### Simulator and metrics

**Fig. 1:**
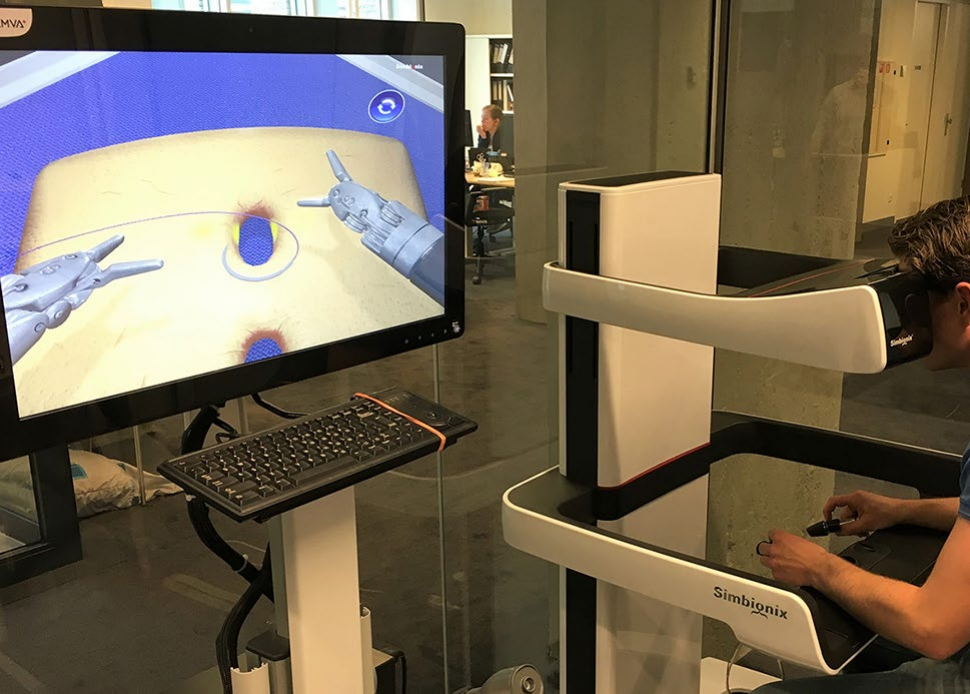
Setup of the RobotiX as used in this study

The RobotiX Mentor platform was used for this study in a standard supplied setup and was installed by 3D Systems. The setup consisted of a tower component and a self-contained unit (the working area) (Fig. 1). The tower held the system monitor and the simulator computer with a keyboard. The self-contained unit consisted of a 3D viewer with head-in sensor, master controllers to steer the robot simulation, ergonomic controls to adjust view height and pedal distance, and the foot pedals to control the clutch, camera, and mono- or bi-polar energy use. The software supplied on the simulator was the “Mentorlearn” which is a web-based simulator curricula management system. For this basic validation study, the “wristed manipulation” and the “vessel energy dissection” tasks were chosen as each task represents a basic and frequently used task from a module. The system recorded over 15 different parameters for each task, which were divided into three domains: movement, safety, and task-specific. The most clinically relevant parameters were selected and are shown in Table 1 with the corresponding parameter definition.

**Table 1:** Parameter definitions as stated by the Mentor learn software

Overall parameters	Definition
Total time	Total time in seconds elapsed between when the user begins the task and starts moving the instruments and when the user finishes or exits the exercise
Path out of view	Total distance traveled by all instruments when not in view in millimeter
Times out of view	Number of times instruments are out of view
Path length left/right	Total distance traveled by the left/right instrument; measured from the clevis not the tool tips in millimeter
Movements left/right	Number of movements of the left/right instrument; a continuous movement of 3 mm or more counts as a movement
Clutch usage	Number of times the clutch is used. One continuous clutch usage will be counted as either: Pedal Clutch as long as the pedal clutch is being pressed. Finger clutch as long as one finger clutch is pressed or both finger clutches are pressed together
Instrument collisions	Number of collisions caused by the instrument shaft wrist and jaws colliding with each other
Task 1: Wristed manipulation	
Accurate targeting	Total time in seconds of instrument collision with the opening of the glass vessel while reaching for a target
Success rate	Percentage of successfully captured targets
Glass vessel movement	Total distance in millimeter of glass vessel movement caused by instrument collision with the vessel
Missed targets	Number of targets that were not captured within the time limit of capturing a target
Task 2: Vessel energy dissection	
Accuracy Energy	(Energy activation time—Time energy applied outside the marks or wrong pedal)/ energy activation time*100
Energy outside marks	Total time in seconds energy is activated outside of the guidance marks
Injury to vessel	Number of times vessel was cut (or damaged by puncturing) not between 2 fully adequate coagulation points
Vessel exposure	Percentage of exposed vessel out of a 3 cm vertical middle section. Total length (cm) of exposed vessel in the middle section/3*100
Total number errors	Sum of the number of errors: Injury to vessel (unsafe cutting). Instrument-instrument collision. Instruments out of view. Wrong energy pedal choice

### Tasks

Tasks representing the most used component tasks in the clinical setting were chosen for validation.

**Fig. 2:**
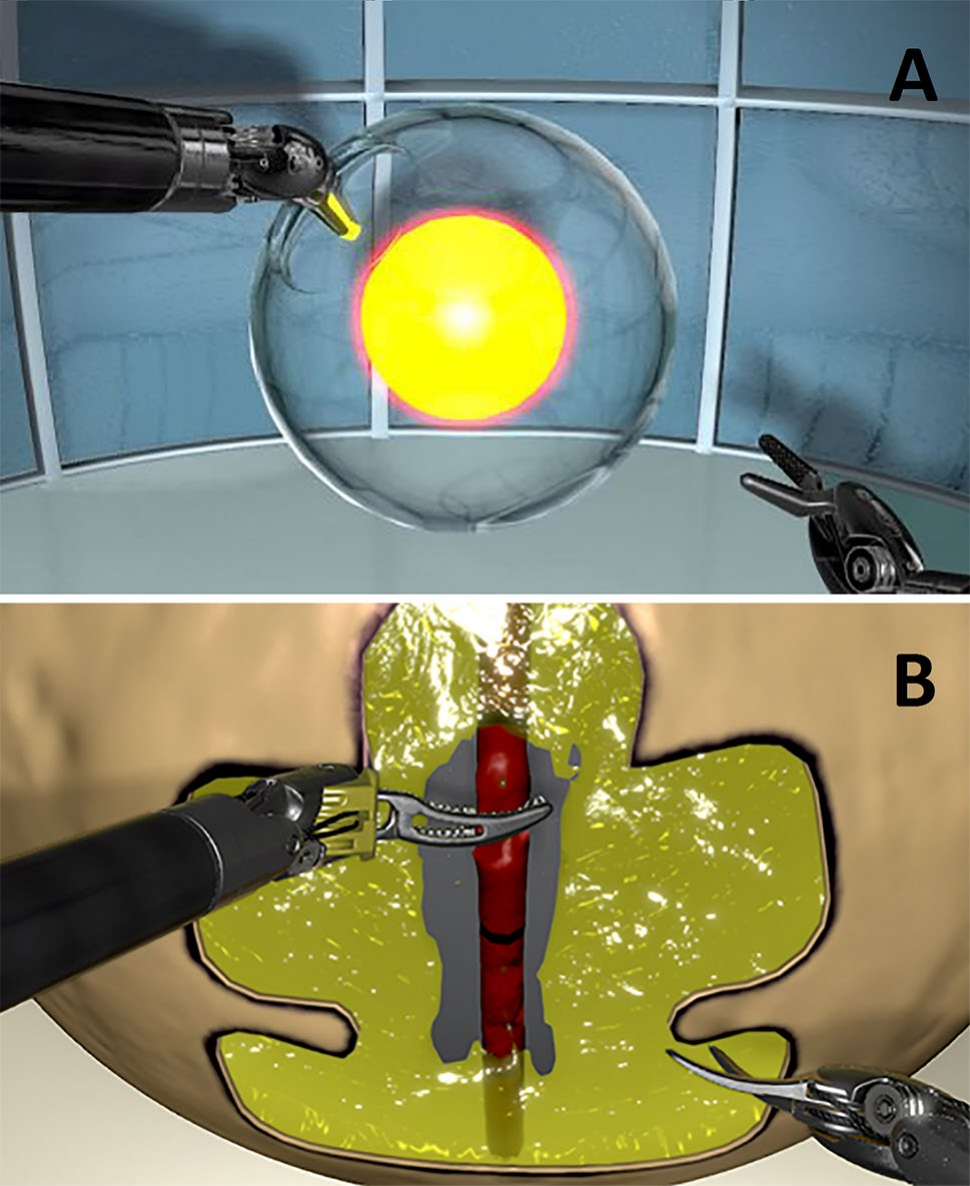
Screenshot of the used tasks. **a** Wristed manipulation (Task 1). **b** Vessel energy dissection (Task 2). Figure provided by 3D-Systems Inc

Task 1: Wristed manipulation (Fig. 2a) is a basic task to encourage the participants to use the wrist capability. The participants started with two needle drivers and a glass sphere in the middle of the screen with one opening in the middle. Inside the glass sphere, a highlighted ball was depicted which must be touched to proceed. After the ball had been touched, the opening switches position, forcing the participant to use their right and left instruments and use different wrist angles. The task was completed when the ball was touched ten times.

Task 2: Vessel energy dissection (Fig. 2b) teaches the participant how to handle delicate tissue and the use of energy through the pedals. The participants started with a grasper and a scissor. Central on the screen, a piece of fatty tissue was depicted with a blood vessel visibly running through this tissue. The participants were instructed to dissect the fatty tissue to expose the vessel and accordingly coagulate the vessel at two targeted points. The task was completed by cutting the vessel between the coagulation points.

### Questionnaire

The questionnaire in this study has been used in the previous validation studies, but has been adjusted to evaluate tasks performed on the RobotiX simulator [21–24]. The questionnaire was divided in two sections (see Supplemental 1). The first section consisted of questions regarding informed consent, demographics, and clinical experience. The questions regarding clinical experience were questions about current profession, years in surgical/gynecologic/urologic training, and number of basic and advanced laparoscopic or robot-assisted procedures performed. Basic procedures were described as non-suturing procedures, such as cholecystectomy and appendectomy. Advanced procedures were defined as procedures with intracorporeal suturing, such as fundoplication or bariatrics. The second section of the questionnaire consisted of three subsections with questions regarding the realism, didactic value and usability of the system, for each separate task, on a five-point Likert scale, with one resulting in strong disagreement, three being the neutral opinion and five representing a strong agreement [25]. There also was an option to fill out ‘No answer’. Realism of the simulator was assessed by questions concerning the perceived realism of the on-screen response, grasper manipulation, tissue response, and on the ability of the task to sufficiently mimic the intended surgical situation in a real-life patient. The didactic value was scored by participant’s opinion on the value of the module to train inexperienced surgeons, to train experienced surgeons, and the value to assess the skills of a trainee. The usability of the system was scored by participants on the user-friendliness of the simulators interface and the appeal of the system to train for this task.

### Protocol

At the entry of the study, participants completed the first section of the questionnaire regarding their demographics and clinical experience. To maintain the response process validity, all participants received the same instruction from one researcher regarding the use of the system. Participants received task-specific instructions with guidance of the written Mentorlearn task explanation. After completion of Task 1 and 2, the participants completed the remainder of the questionnaire. To assess the content source of validity (does the measured content reflects the characteristic it intends to measure), the subsections of the questionnaire were divided into three sections concerning the realism, didactic value and usability. The perceived realism was assessed, because a simulated task is desired to have a high-level correspondence to the clinical setting with similar instrument and tissue handling. Accordingly, the didactic value was assessed to determine the perceived value of each task to train participants of different levels of expertise. Finally, the usability of the system for the specific task was attained, to determine the perceived value of this system as a method to train the performed tasks. The main outcome of these values is dependent from the expert group as they contain the training and clinical experience. However, novices, residents, and laparoscopic experienced surgeons are the future robot-assisted trainees and were, therefore, included in the assessment of the simulated tasks.

The relation to other variables validity evidence consists of the capability of the assessment outcome to differentiate between skill levels of the users. The skill level of interest usually is related to the level of surgical experience such as experts or novices. Performances outcomes that are significantly different between novices and experts can be identified as such by the researched simulator, which determines the relation to other variables’ validity evidence. The performance score of each participant was calculated after each task was performed once, without any previous experience on the performed tasks. Participants received a maximum of 20 min for each task. The performance parameters were saved for construct analysis. Accordingly, to determine the consequence of the test validity evidence, a composite proficiency score was calculated for each group. Subsequently, with the proficiency score, a pass/fail cut-off value was calculated.

### Statistical analysis

#### Content and relationship to other variables

To determine the difference between the experience groups regarding the questionnaire answers and performance outcomes, independent *t* test analyses were used to determine significant differences. Metrics resulting in a significant difference between the robotic experienced and novice group and in favor of the robotic experienced group were included for the calculation of a composite score ranging from 0 to 100. The composite score was calculated by linear normalization of the included parameters. The composite score outcomes were compared between groups using the independent *t* test.

#### Consequences

For the determination of a pass/fail standard, the composite scores were compared with the contrasting group method using the calculation model by Jorgensen et al. [26]. To compare the usability of the pass/fail score from the contrasting group method, benchmark scores were also calculated from the 25th percentile of the robotic experienced participants. Benchmarks based on the 25th percentile were addressed and used in the previous studies [13, 27]. All *p* values < 0.05 were considered statistically significant. The analysis was performed using the Statistical Package for Social Sciences (SPSS) version 22 (IBM Corp., Armonk NY).

## Results

This study enrolled 61 participants, which resulted in 27 novices, 21 laparoscopic, and 13 robotic experienced participants. The novice participants consisted of medical students without any clinical experience or training, with a mean age of 24 years. Participants in the laparoscopic experienced group had a mean age of 36 years and consisted of residents in training for 3 (*n* = 3), 4 (*n* = 7), and 5 years (*n* = 4) and seven specialized surgeons. The disciplines in the laparoscopic experienced group contained urology (*n* = 3), gynecology (*n* = 10), surgery (*n* = 5), and pediatric surgery (*n* = 3). The laparoscopic group had a median clinical laparoscopic experience of 1–5 years with a median performance of 51–100 basic and 6–10 advanced procedures. The robotic experienced group had a mean age of 44 years and consisted of ten specialized surgeons and three resident in training in the 4th, 5th, and 6th years. The robotic experienced participants were mostly from the urologic discipline (*n* = 10) and the remaining three participants were surgeons. The robotic experienced participants consisted of one participant with less than ten procedures of experience with basic robot-assisted procedures but having performed 11–20 advanced procedures robot-assisted. Four participants had basic robot-assisted experience ranging from 11 to 30 procedures, one participant with 51–100 procedures, and seven participants with > 100 procedures of experience.

### Realism, didactic value, and usability (content)

#### Task 1

The opinion scores on realism, didactic value, and usability are shown in Table 2. Overall realism, didactic value, and usability were scored positively by all groups. Statistically significant differences in opinions were found for the robotic experienced participants compared to the novices on the overall realism (3.3 versus 3.9, *p* = 0.031), on-screen response of instruments (3.5 versus 4.3, *p* = 0.038), physical manipulation of the graspers (3.0 versus 3.8, *p* = 0.005), and appeal as a tool for this task (3.7 versus 4.4, *p* = 0.005). The realism of the wristed manipulation task received the highest scores for the ‘on-screen response of the tools’ (robotic 3.5, laparoscopic 4.2, and novice 4.3) and the lowest scores for the realism of the ‘tissue behavior’ (robotic 2.9, laparoscopic 3.1, and novice 3.5). The mean overall didactic value score was positive by all participants, although the lowest scores were for the didactic value of the RobotiX simulator as a tool to train surgeons (robotic 3.0, laparoscopic 3.6, and novice 3.3) and the highest scores in terms of ability to train novices (robotic 3.9, laparoscopic 4.2, and novice 4.3) which corresponds with the purpose of this task. The usability of the RobotiX scored a mean of 4.1, with participants rating the simulator interface of the RobotiX the highest (robotic 3.8, laparoscopic 4.3, and novice 4.1).

**Table 2:** Mean (SD) scores on the realism, didactic value, and usability of Task 1

Task 1: Wristed manipulation	Robotic experienced	Laparoscopic experienced	Novices
	*n* = 13	*n* = 21	*n* = 27
Realism overall score	3.3 (0.9)	3.7 (0.7)	3.9 (0.6)
On-screen response of instruments	3.5 (1.1)	4.2 (0.7)	4.3 (0.7)
Physical manipulation of graspers	3.0 (1.1)	3.8 (1.0)	4.0 (0.9)
Task sufficiently realistic	3.6 (0.9)	3.6 (1.0)	3.8 (0.9)
Behavior of the tissue	2.9 (1.0)	3.1 (1.2)	3.5 (0.8)
Didactic value overall score	3.6 (0.8)	4.0 (0.6)	3.9 (0.6)
To train novices	3.9 (0.9)	4.2 (0.9)	4.3 (0.6)
To train surgeons	3.0 (1.1)	3.6 (1.4)	3.3 (0.9)
Assessment of a trainee	3.9 (0.9)	4.1 (0.6)	3.8 (0.7)
Usability overall score	3.8 (0.7)	4.3 (0.5)	4.2 (0.7)
Simulator interface	3.8 (0.8)	4.3 (0.6)	4.1 (0.8)
Appeal as a tool for this task	3.7 (0.7)	4.3 (0.6)	4.4 (0.7)

Data in this table represent mean opinion values and standard deviations (SD). Statistical differences were calculated with the independent t tests between each group (*R*  robotic experienced, *L* laparoscopic experienced, and *N* novices). *p* values of < 0.05 were considered significant

#### Task 2

Task 2 received overall positive scores by all participants (mean realism 3.7, didactic value 3.9, and usability 4.1), as shown in Table 3. Statistically significant differences in opinion scores were found between robotic experienced and novices for the realism of the on-screen response of instruments (*p* = 0.027), usability overall (*p* = 0.017), and the appeal as a tool for this task (*p* = 0.026). Realism was scored lowest for the tissue behavior (robotic 3.2, laparoscopic 3.5, and novice 3.3) with a strong consensus between the groups. The highest scores for realism were found for on-screen response (robotic 3.8, laparoscopic 4.2, and novice 4.3). Similar to Task 1, the didactic value of the simulator to train surgeons scored the lowest (robotic 3.4, laparoscopic 3.5, and novice 3.4). Didactic value of the simulator to train novices was rated highly positive by all groups (robotic 4.2, laparoscopic 4.2, and novice 4.3). Items on user-friendliness of the interface and the appeal of the RobotiX received both a good positive overall score of 4.0.

**Table 3:** Mean (SD) scores on the realism, didactic value and usability of Task 2

Task 2: Vessel energy dissection	Robotic experienced	Laparoscopic experienced	Novices
	*n* = 13	*n* = 21	*n* = 27
Realism overall score	3.5 (0.8)	3.9 (0.5)	3.8 (0.6)
On-screen response of instruments	3.8 (0.7)	4.2 (0.6)	4.3 (0.5)
Physical manipulation of graspers	3.3 (0.9)	4.0 (0.9)	3.9 (0.9)
Task sufficiently realistic	3.8 (0.7)	3.8 (0.6)	3.8 (0.8)
Behavior of the tissue	3.2 (1.2)	3.5 (0.9)	3.3 (1.0)
Didactic value overall score	3.8 (0.8)	4.0 (0.8)	3.9 (0.5)
To train novices	4.2 (0.7)	4.2 (1.0)	4.3 (0.6)
To train surgeons	3.4 (1.1)	3.5 (1.4)	3.4 (0.9)
Assessment of a trainee	3.9 (0.9)	4.1 (0.8)	3.8 (0.7)
Usability overall score	3.8 (0.4)	4.2 (0.6)	4.2 (0.6)
Simulator interface	3.8 (0.4)	4.1 (0.7)	4.2 (0.6)
Appeal as a tool for this task	3.7 (0.5)	4.2 (0.6)	4.2 (0.7)

### Relation to other variables

#### Task 1

The mean performance outcomes per group are shown in Table 4. Robotic experienced participants outperformed novices for the parameters movements left (82 versus 109, *p* = 0.009), movements right (86 versus 104, *p =* 0.009), traveled path left (1149 mm versus 1417 mm, *p =* 0.020), and total time (114 s versus 162 s, *p =* 0.040). For the accurate targeting, robotic experienced users spent less time in collision with the glass vessel opening compared to the laparoscopic and novice groups (mean 8.1 s, 11.4 s and 12.7 s respectively). Additionally, the robotic experienced group had higher mean scores for the clutch usage, compared to the laparoscopic and novice group (6.1, 3.6, and 2.4 times respectively). However, none of these parameters were able to show statistically significant differences.

**Table 4:** Mean (SD) performance outcomes per group on Task 1

Task 1: Wristed manipulation	Robotic experience	Laparoscopic experience	Novice	*p* values		
	*n* = 13	*n =* 21	*n =* 27	*R* vs *N*	*L* vs *N*	*R* vs *L*
Movement						
Movements left	82 (19)	105 (49)	109 (42)	*0.009*	0.758	0.071
Movements right	86 (17)	104 (47)	109 (35)	*0.009*	0.713	0.112
Path left	1149 (129)	1322 (437)	1417 (535)	*0.020*	0.513	0.102
Path right	1168 (174)	1198 (538)	1133 (611)	0.782	0.700	0.815
Safety						
Missed targets	0.5 (0.7)	0.9 (1.8)	0.6 (1.0)	0.763	0.391	0.392
Instrument collisions	0.0 (0.0)	0.0 (0.2)	0.0 (0.2)	0.495	0.859	0.440
Success rate	95 (7)	91 (18)	94 (10)	0.727	0.391	0.392
Path out of view	25 (67)	10 (29)	13 (32)	0.465	0.728	0.387
Times out of view	0.9 (1.0)	0.7 (0.8)	0.9 (1.3)	0.861	0.567	0.404
Task specific						
Total time	114 (54)	150 (63)	162 (72)	*0.040*	0.551	0.096
Accurate targeting	8.1 (4.0)	11.4 (7.5)	12.7 (10.8)	0.143	0.628	0.157
Glass movement	131 (146)	130 (145)	115 (106)	0.701	0.675	0.993
Clutch usage	6.1 (8.8)	3.6 (6.0)	2.4 (4.3)	0.169	0.406	0.339

Data in this table represent mean values and standard deviations (SD). Statistical differences were calculated with the independent *t* tests between each group (*R* robotic experienced, *L* laparoscopic experienced, and *N =* novices). *p* values of < 0.05 were considered significant (displayed in italics)

#### Task 2

The mean performance scores of Task 2 are shown in Table 5. Statistically significant differences between robotic experienced and novice participants were found for the parameters movements right (132 versus 179, *p =* 0.021), path out of view (19 mm versus 0 mm, *p =* 0.016), and total time (147 s versus 265 s, *p* < 0.001). The laparoscopic experienced participants performed similar to the novice participants, showing no statistically significant differences between these groups except for the path right parameter (1257 mm versus 1749 mm, *p =* 0.040). Between the robotic and laparoscopic experienced participants, statistically significantly differences were found for the parameters movements left (72 versus 109, *p =* 0.043), path out of view (19 mm versus 0 mm, *p =* 0.014), total time (147 s versus 239 s, *p =* 0.003), and clutch usage (2.2 versus 0.7 times, *p =* 0.021).

**Table 5:** Mean (SD) performance outcomes per group on Task 2

Task 2: Vessel energy dissection	Robotic experience	Laparoscopic experience	Novice	*P* values		
	*n =* 13	*n =* 21	*n =* 27	R vs N	L vs N	R vs L
Movement						
Movements left	72 (37)	109 (58)	105 (59)	0.069	0.802	*0.043*
Movements right	132 (35)	167 (92)	179 (88)	*0.021*	0.635	0.207
Path left	561 (331)	859 (568)	754 (539)	0.245	0.516	0.097
Path right	1394 (561)	1257 (711)	1749 (864)	0.186	*0.040*	0.562
Safety						
Injury to vessel	0.2 (0.4)	0.4 (0.6)	0.2 (0.5)	0.838	0.137	0.110
Energy outside marks	0.5 (1.2)	0.0 (0.0)	1.0 (2.6)	0.559	0.065	0.147
Total number errors	7.7 (7.9)	7.4 (6.6)	5.3 (5.0)	0.347	0.204	0.927
Instrument collisions	4.0 (4.9)	6.6 (6.6)	4.8 (4.9)	0.624	0.283	0.228
Path out of view	19 (24)	0 (0)	0 (2)	*0.016*	0.384	*0.014*
Task specific						
Total time	147 (50)	239 (92)	265 (111)	< *0.001*	0.384	*0.003*
Vessel exposure	96 (3)	95 (3)	95 (3)	0.550	0.448	0.226
Accuracy energy	79 (24)	87 (22)	79 (24)	0.967	0.255	0.366
Clutch usage	2.2 (2.4)	0.7 (1.3)	1.7 (4.2)	0.696	0.281	*0.021*

Data in this table represent mean values and standard deviations (SD). Statistical differences were calculated with the independent *t* tests between each group (*R*  robotic experienced, *L*  laparoscopic experienced, and *N *  novices). *p* values of < 0.05 were considered significant (displayed in italics)

### Composite performance score

**Fig. 3:**
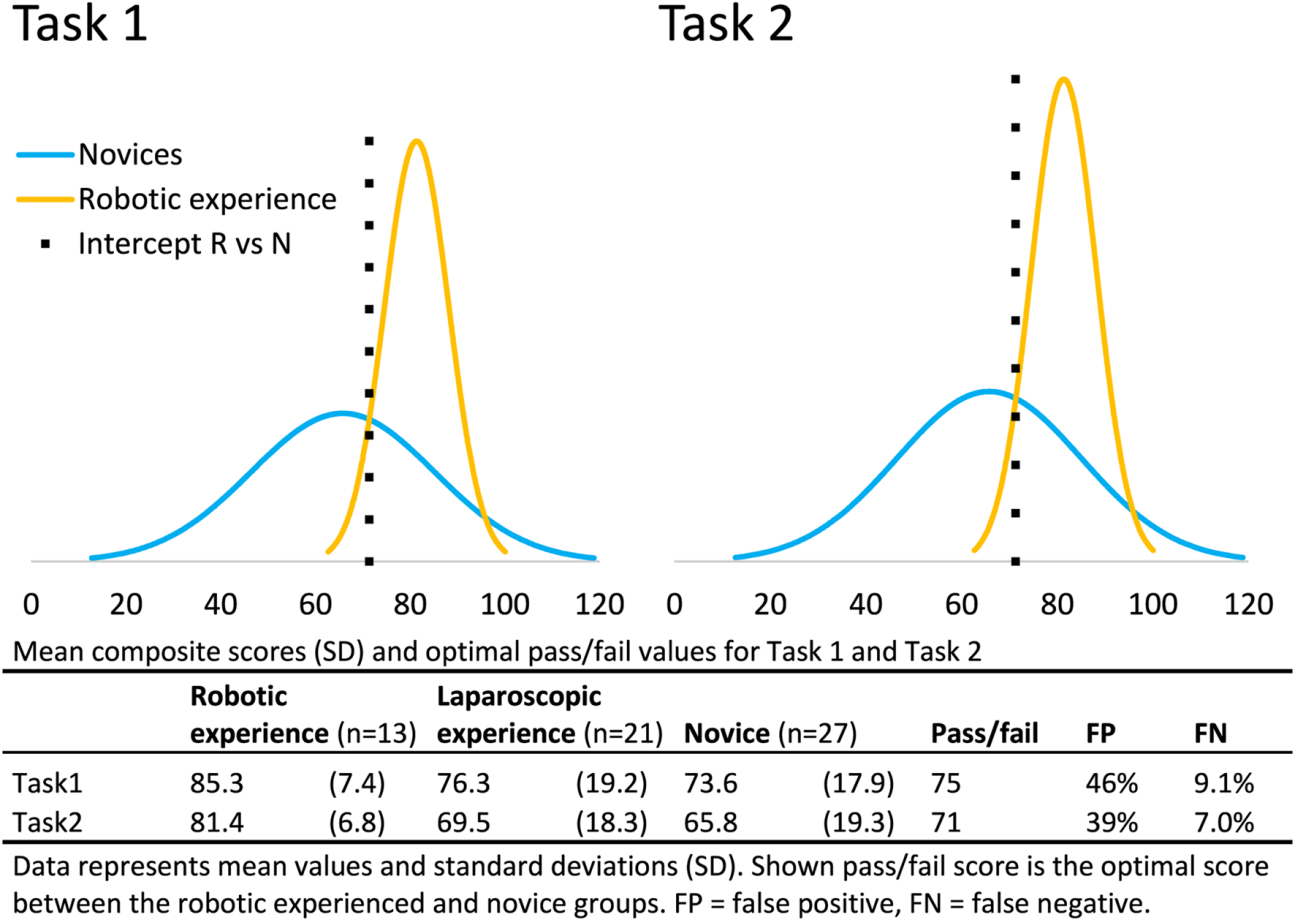
Mean (SD) composite score outcomes of all groups and the contrasting group analysis between the robotic experienced and novice groups

The composite score for Task 1 was composed of the parameters ‘number of movements left’, ‘number of movements right’, ‘path left,’ and ‘total time’ as these were statistically significant different between the robotic experienced and novice participants. For Task 2, the composite score was calculated with the parameters ‘number of movements right’ and ‘total time’. The parameter ‘path out of view’ was not included, because the robotic experienced participants were outperformed by the novice and laparoscopic experienced group. The mean composite scores calculated for Task 1 and 2 are shown in Fig. 3. Comparing the mean composite scores between the groups resulted in statistically significant differences between robotic experienced participants and novices for Task 1 and 2 (85.3 versus 73.6, *p =* 0.006 and 81.4 versus 65.8, *p =* 0.001, respectively). Accordingly, a pass/fail cut-off score of 75 and 71 was calculated for both tasks. The dotted line (Intercept) represents the ideal pass/fail score with the lowest percentage of novices being scored as competent (false positive) and robotic experienced participants being scored as inadequate (false negative). The calculated pass/fail scores showed a theoretical false-positive/false-negative score of 46%/9.1% for Task 1 and 39%/7.0% for Task 2.

### Benchmark

To compare the usability of the contrasting group method with the pass/fail score to the benchmark scores derived from the 25th percentile, an additional analysis was performed. This resulted in the benchmark values showed in Table 6 with the according percentage of novices and laparoscopic experienced participants passing that benchmark. For Task 1, the parameters missed targets, instrument collisions, success rate, path out of view, times out of view, and clutch usage showed to be least specific in distinguishing between novices and robotic experienced participants, because a substantial part of the novices passed the benchmark. The laparoscopic experienced participants showed a better percentage of participants passing the benchmark compared to the novices for the parameters movements right (29% versus 11%), total time (5% versus 0%), and composite score (29% versus 7%). The benchmark values of Task 2 showed that the parameters injury to vessel, energy outside marks, path out of view, accuracy of energy, and clutch usage were unspecific as they have a high percentage of novices passing the benchmark. The laparoscopic experienced participants showed a higher percentage passing the benchmark compared to novices for the parameters path right (67% versus 22%), energy outside marks (100% versus 82%), and total time (5% versus 0%).

**Table 6:** Benchmark scores and percentage of novices (*N*) and laparoscopic experienced participants (*L*) passing the benchmark

Task 1: Wristed manipulation	Bench mark	*N* (%)	*L* (%)	Task 2: Vessel energy dissection	Bench mark	*N* (%)	*L* (%)
Movement							
Movements left	67	7	14%	Movements left	41	11	14
Movements right	76	11	29	Movements right	101	11	14
Path left	1032	22	24	Path left	265	19	10
Path right	1032	30	33	Path right	1089	22	67
Safety							
Missed targets	0	70	52	Injury to vessel	0	85	92
Instrument collisions	0	96	95	Energy outside marks	0	82	100
Success rate	100	70	52	Total number errors	2	22	19
Path out of view	0	63	67	Instrument collisions	0	15	14
Times out of view	0	52	48	Path out of view	0	96	100
Task specific							
Total time	78	0	5	Total time	109	0	5
Accurate targeting	5	11	24	Vessel exposure	98	22	10
Glass movement	45	26	33	Accuracy energy	100	44	52
Clutch usage	1	41	48	Clutch usage	1	63	67
Composite score	90	7	29	Composite score	85	7	10

Data in this table represent the 25th percentile benchmark based on the performance of the robotic experienced participants. *N *  novices, *L* laparoscopic experienced

## Discussion

With this study, we investigated the evidence of validity on multiple levels of Messick’s framework [19]. The content, response process, relation to other variables, and consequences of the test have been assessed. This study found a pass/fail score based on the contrasting groups analysis of 75 and 71 for the wristed manipulation and the vessel energy dissection task, respectively. However, the usability of this pass/fail score showed to be limited due to the high percentage of false-positive outcomes. The alternative benchmark analysis resulted in usable target scores for novices and laparoscopic experienced participants. These results can be used during future surgical training assessment.

The previous studies were performed to validate the RobotiX and the available tasks [9–14]. Validity for the RobotiX system itself was determined by Hertz et al., although it does not specifically determine task-specific validity evidence [10]. A larger study by Whitaker et al. has validated multiple modules of the RobotiX including the currently studied tasks [9]. However, these outcomes were specified per complete module, which made it unable to compare the construct outcomes. Of the previous validity studies, only the study by Hovgaard et al. used the modern validity framework which is considered the new standard of evaluating validity of simulation and assessment [12, 28, 29]. Therefore, a major strength of our study is the use of this framework, combined with a relatively large study population, compared to the previous performed studies (61 versus a maximum of 46 participants). The inclusion of different experience levels and surgical specialties with the addition of the laparoscopic ‘target’ group further strengthened the input of the content. The study by Watkinson et al. previously determined benchmark scores of the wristed manipulation and other similar tasks [13]. The benchmark outcomes were similar to the results found for Task 1 in this study with a benchmark score for distance by camera, instrument collisions, and times out of view of zero. Additionally, the benchmark for the parameters path length left (915.5 mm versus 1032 mm) and path length right (959.3 mm versus 1032 mm) were comparable to this study. However, the time benchmark score in this study was found to be substantial lower (78 s versus 105.1 s). This is likely caused by differences in the robotic experienced groups. In the study by Watkinson et al., the robotic experienced group performed a mean of 26.7 (range 1–80) robot-assisted procedures, whereas in this study, 7 out of the 13 robotic experienced participants had more than 100 procedures of experience. The effect of better robotic experienced participants is also shown in the percentage of novices passing the time benchmarks score, because 0% passed the 78 s benchmark compared to 35% in the study by Watkinson et al. By demanding a faster task completion time, participants are likely to be less focused on completing the task utilizing the required skills and caution for safety. Therefore, the time benchmark found by Watkinson et al. could be more favorable for the assessment of a trainee.

This study showed the evidence of validity on the wristed manipulation and energy vessel dissection task. However, robotic experienced users scored the grasper manipulation less than favorably, with a mean of 3.0 and 3.3 for Task 1 and 2, respectively. This is most likely because of the different type of controller setup in the RobotiX compared to the Davinci system. Both laparoscopic and robotic experienced users scored the tissue behavior of the wristed manipulation task low, which is explained by participants noticing the surrounding to be un-realistic and fragile, not mimicking the clinical setting. Also, the energy vessel dissection task received low scores on tissue realism, which is explained by the vast clinical experience of participants in the robotic experienced group. Second, the RobotiX system uses a different type of 3D viewer that required users to adjust the lenses to the width of their eyes, and some participants had trouble fine-tuning these settings, possibly affecting their view and consequently their performance.

The contrasting group method to determine a credible pass/fail score in this study was found to be limited due to the high percentage of false positives. The main cause of this limitation was the high variability in performance of the novice group which led to a high standard deviation. Additionally, an analysis to determine possible novice subgroups and regarding the most experienced robotic group was performed (not shown), but did not result in any new significant outcomes. Another factor affecting the pass/fail score was the limited number of parameters showing construct to be included in the composite score. A possible explanation for the lack of parameters showing construct is the short duration of the task, which could make it more difficult to prove significant differences. A larger group could show significant differences, although the question remains whether that would be clinically relevant. This was a relatively easy task, to get acquainted with robotic surgery, which may not be as difficult as expected and no robotic expertise is needed for a good result of this task. Therefore, the contrasting group analysis was shown to be unfit for the assessment of novices in this study.

Virtual Reality simulators are designed to create a safe didactic training setting, which, consequently, leads to simulators aiming at learning fine instrument movements, soft-tissue handling, and awareness of the surrounding environment. This is learned by guidance, warning, and addressing users at errors on the slightest occasion. Consequently, this teaches trainees to perform the task with more care than would perhaps be necessary in a clinical setting. However, increased clinical experience could allow a participant to perform a basic task quicker, and possibly less precise in a simulated setting. Therefore, the amount of clinical experience could work as a confounder on some of these parameters. An example found in this study is the path out of view parameter from Task 2, where the novices and laparoscopic experienced outperformed the robotic experienced group. This is most likely because the robotic experienced participants were more aware of their instruments and, therefore, reacted instinctively when their instruments were out of view and did not focus on this assessment parameter. To eliminate this effect, a repetitive exercise study is required to further determine the optimal simulator performance scores and possible learning curve of novice participants.

## Conclusion

With this study, validity evidence has been gathered for the wristed manipulation and energy vessel dissection tasks of the RobotiX simulator. The didactic value to train inexperienced surgeons was scored high, corresponding to the goal of these two basic tasks. Aspects that could require additional attention in the further development are instrument handling and tissue behavior. The calculated pass/fail cut-off scores showed to be limited in the assessment of novice trainees. However, the provided benchmark scores showed to be adequate to assess novice and laparoscopic experienced trainees. Therefore, these results can be used for the assessment of trainees of these basic robot-assisted skills.

## Electronic supplementary material

Below is the link to the electronic supplementary material.

Electronic supplementary material 1 (DOCX 55 kb)

## Data Availability

The data sets used and analyzed in this study are available from the corresponding author on reasonable request.
